# Pain Is in the Timing: The Emerging Importance of Phase‐Based EEG Connectivity

**DOI:** 10.1002/ejp.70325

**Published:** 2026-07-10

**Authors:** Enrico De Martino

**Affiliations:** ^1^ Center for Neuroplasticity and Pain (CNAP), Department of Health Science and Technology Aalborg University Gistrup Denmark

This journal recently published a paper by Han et al., entitled ‘*Predicting Pain: Electroencephalography Signatures of Neural Integration during Experimental Tonic Thermal Pain*’ (Han et al. 2026). Their findings suggest that phase‐based EEG features provide more information about tonic pain than conventional power measures. Although much of the EEG pain literature has focused on changes in oscillatory power, Han et al. showed that measures of phase synchrony and phase‐based cross‐frequency coupling discriminated painful from non‐painful states more accurately than power‐based metrics. The addition of amplitude‐related features did not improve classification and, in some analyses, reduced performance. These results suggest that the temporal coordination of neural activity may carry information about pain that is not captured by changes in signal amplitude alone.

The study adds to a growing body of work indicating that pain is associated with changes in large‐scale cortical interactions rather than solely with alterations in local activity. From this perspective, EEG markers based on connectivity may provide information that complements traditional spectral measures. Phase relationships are particularly relevant because they reflect the temporal organization of activity across distributed neural populations and may offer insight into the network dynamics underlying pain processing.

The findings of Han et al. are consistent with results obtained using different approaches. In an experimental pain model, transcranial magnetic stimulation combined with electroencephalography showed that acute pain reduced alpha‐band inter‐trial coherence in parietal‐occipital regions, and these changes correlated with individual pain sensitivity (De Martino et al. 2024). Notably, the effects were more evident in phase‐based measures than in conventional oscillatory power. Likewise, in a study of chronic pain patients undergoing repetitive transcranial magnetic stimulation, connectivity patterns derived from the debiased weighted phase lag index differentiated treatment responders from non‐responders (De Martino et al. 2025). Although these studies addressed different questions and populations, they point to a common role of phase synchronization in pain‐related brain dynamics.

Across experimental tonic pain, acute pain models and chronic pain, phase‐based measures appear sensitive to changes in pain processing. Whether assessed through inter‐site phase clustering, inter‐trial coherence or phase lag‐based connectivity metrics, these approaches consistently highlight the importance of temporal coordination between neural populations.

Han et al. provide further support for the idea that the timing of neural activity may be as important as its magnitude for understanding pain‐related brain function. The development of EEG‐based pain biomarkers may therefore benefit from greater attention to measures of neural synchronization and network connectivity, alongside traditional analyses of oscillatory power.

## Funding

The author has nothing to report.

## Conflicts of Interest

The author declares no conflicts of interest.

## Linked Articles

This article is linked to Torrado‐Carvajal and Loggia papers. To view this article, visit https://doi.org/10.1002/ejp.70324.
